# Natural products and immune-cell responses in osteoarthritis: mechanisms, evidence maturity, and translational gaps

**DOI:** 10.3389/fimmu.2026.1883774

**Published:** 2026-07-10

**Authors:** Jinhu Jia, Yi Zhang, Weizheng Wang, Yangfan Qi, Jinliang Gao

**Affiliations:** 1 Changchun University of Chinese Medicine, Changchun, China; 2 Department of Rheumatology and Immunology, Affiliated Hospital of Changchun University of Chinese Medicine, Changchun, China; 3 Jiaozuo People’s Hospital, Jiaozuo, China

**Keywords:** immune cells, immunomodulation, natural products, osteoarthritis, synovitis, translational medicine

## Abstract

Osteoarthritis (OA) is increasingly recognized as an immune-associated whole-joint disorder characterized by chronic low-grade inflammation, which contributes to joint degeneration, structural deterioration, and persistent pain. Innate and adaptive immune cells, including macrophages, T cells, neutrophils, and mast cells, participate in OA pathogenesis by releasing pro-inflammatory cytokines, reactive oxygen species, and matrix-degrading enzymes, as well as by interacting with chondrocytes, synovial fibroblasts, and subchondral bone cells. Natural products, because of their multi-target pharmacological properties, have emerged as potential modulators of this complex immune microenvironment. This review critically appraises current evidence on natural-product interventions modulating OA-associated immune-cell responses, with particular emphasis on mechanistic evidence, evidence maturity, and translational potential. It further compares evidence across immune-cell populations to identify shared mechanisms, population-specific differences, and key translational gaps. Macrophage- and T-cell-associated responses have the most developed evidence base, with relatively consistent evidence supporting modulation of M1-like/M2-like macrophage phenotypes and the T helper 17 (Th17)/regulatory T (Treg) cell balance. Neutrophil- and mast-cell-associated responses represent emerging or auxiliary areas of evidence, as these immune-cell populations may amplify inflammation and pain through reactive oxygen species production, neutrophil extracellular trap formation, degranulation, and inflammatory mediator release. Evidence for B cells, dendritic cells (DCs), and natural killer (NK) cells remains limited; therefore, these immune-cell populations should currently be regarded as potential research directions rather than established therapeutic targets. Most available data derive from *in vitro* and animal studies, whereas clinical evidence is largely restricted to peripheral immune-marker changes and short-term symptom improvement. Future studies should integrate local joint immune profiling, functional validation, and stratified clinical designs to clarify the translational potential of natural products in OA immunomodulation.

## 1 Introduction

Osteoarthritis (OA) has traditionally been viewed as a chronic joint disease driven by mechanical stress, cartilage wear, and age-related degeneration. However, this concept does not fully explain the pathological heterogeneity of OA, the frequent presence of synovitis, or the persistent pain experienced by many patients (1). Increasing evidence indicates that OA is not merely a “wear-and-tear” disease. Instead, it is a whole-joint disorder involving the cartilage, synovium, subchondral bone, infrapatellar fat pad, and neuro-immune axis. In this context, immune-associated chronic low-grade inflammation is not simply a secondary consequence of tissue damage. It may contribute to disease progression, structural deterioration, and symptom persistence (2–4). Within the osteoarthritic joint, damaged cartilage, synovial tissue, and subchondral bone release damage-associated molecular patterns (DAMPs) and other endogenous danger signals. These molecules activate pattern-recognition receptor-related pathways and promote local inflammatory responses in the synovium and cartilage (5, 6). Innate immune cells, including macrophages, neutrophils, and mast cells, subsequently secrete pro-inflammatory cytokines, chemokines, reactive oxygen species (ROS), and proteolytic enzymes. Key mediators include interleukin-1β (IL-1β), tumor necrosis factor-α (TNF-α), and interleukin-6 (IL-6). Collectively, these inflammatory signals promote synovial inflammation, cartilage matrix degradation, subchondral bone remodeling, and pain sensitization (7–11). Adaptive immune cells, particularly T and B lymphocytes, may further amplify local inflammation and disrupt immune homeostasis. For example, an imbalance between Th17/Treg cells, B-cell infiltration, and related cytokine networks have been associated with specific OA phenotypes (12, 13). Therefore, although OA differs from classical autoimmune diseases such as rheumatoid arthritis (RA), immune dysregulation is clearly involved in its pathogenesis (14, 15). However, OA and RA differ substantially in their immunopathological mechanisms. Immune responses in RA are typically associated with systemic autoimmunity, autoantibody production, persistent synovitis, and pannus formation. In contrast, immune activation in OA is generally lower in intensity, more heterogeneous, and more closely related to local tissue injury, mechanical stress, metabolic disturbance, synovitis, and pain sensitization. Therefore, natural products in OA should not be interpreted as immunosuppressive or disease-modifying agents analogous to those used in RA. Rather, they should be considered potential interventions that may modulate local inflammatory amplification, oxidative stress, immune-cell interactions with joint structural cells, and phenotype-related immune imbalance.

Natural products have attracted increasing attention in basic, translational, and clinical OA research because of their multi-target pharmacological properties. Many natural compounds exhibit anti-inflammatory, antioxidant, chondroprotective, and analgesic effects (16, 17). Previous reviews have commonly classified natural products according to chemical category, inflammatory pathway, or chondroprotective activity. These approaches are useful for summarizing pharmacological effects, but they often do not clearly distinguish general anti-inflammatory activity from cell-specific immunomodulation. For example, Lee et al. summarized natural compounds affecting inflammatory pathways in OA, with emphasis on oxidative stress, inflammatory mediators, and related signaling pathways. Within such a framework, reductions in IL-1β, TNF-α, IL-6, or matrix metalloproteinases (MMPs), or inhibition of nuclear factor-κB (NF-κB)/NLRP3 signaling, may support anti-inflammatory or chondroprotective effects. However, without evaluating macrophage phenotypes or macrophage-specific mechanisms, these findings cannot confirm local macrophage regulation (18). Similarly, Maouche et al. reviewed bioactive compounds in OA according to molecular mechanisms and therapeutic effects. However, changes in overall inflammatory markers or peripheral immune indices are insufficient to demonstrate local immune-cell modulation in the synovium or synovial fluid (19). Therefore, unlike previous reviews that mainly summarize compounds, pathways, or disease-related outcomes, the distinct contribution of this review is the application of an immune-cell-type-centered evidence maturity framework. This framework stratifies evidence related to different immune-cell populations across six dimensions: pathological relevance, level of evidence, local joint evidence, functional validation, intervention standardization, and translational potential. Importantly, this framework does not assume that natural products act exclusively or directly on immune cells. Instead, it evaluates the extent to which immune cells may participate in the protective effects of natural products and whether the available evidence is sufficient to support cell-specific immunomodulation. By applying this approach, we aim to provide a more precise assessment of the current evidence, distinguish mature from emerging immune-cell-associated evidence, and identify key knowledge gaps to guide future mechanistic studies and clinical trial design.

## 2 Evidence assessment framework

This review adopts a narrative review approach to critically evaluate evidence related to natural-product interventions and immune-cell regulation in OA. The interventions discussed include purified compounds, standardized extracts, and natural-product preparations with clearly described active components. Purified compounds and standardized extracts are generally more suitable for mechanistic interpretation because their composition and biological effects can be more clearly defined. In contrast, complex formulations and combination interventions may provide useful translational signals, but they should be interpreted cautiously. When local immune assessment or functional validation is lacking, such interventions should not be considered confirmatory evidence of cell-specific immunomodulation. To provide an overview of natural-product interventions in OA, **Table 1** summarizes the major categories, sources, and intervention forms of representative natural products. To evaluate the maturity of evidence related to each immune-cell population, this review uses six predefined dimensions. First, we assessed pathological relevance, defined as whether a given immune-cell type is involved in OA-associated inflammation, cartilage degeneration, synovial changes, subchondral bone remodeling, or pain sensitization. Second, we considered the level of evidence, including whether findings were limited to *in vitro* experiments or had progressed to animal models or human studies. Third, we examined local joint immune evidence, particularly whether immune changes were assessed in the synovium, synovial fluid, or osteochondral unit, rather than being limited to peripheral blood. Fourth, we evaluated functional validation, including whether causal relationships were tested through cell depletion, adoptive transfer, pathway blockade, or cell-specific analyses. Fifth, we considered intervention standardization, including the clarity of natural-product composition, dosage, purity, formulation characteristics, route of administration, and batch consistency. Sixth, we assessed translational potential, including the availability of human data and evidence of symptom improvement, imaging outcomes, structural endpoints, or clinically relevant biomarkers.

**Table 1:** Literature overview of natural products modulating immune cells in osteoarthritis.

Immune-cell population	Representative natural products	Main mechanism/pathway	Evidence type	Local joint evidence	Functional validation	Translational comment
Macrophages	Liquiritin; Polydatin; THSG; Quercetin; Apigenin; Nystose; Caesalpinia sappan extract; Puerarin	M1→M2 shift; Rap1/PI3K/Akt; NF-κB;TRPV1/P2X7/NLRP3; TRPM7-mTOR	*In vitro* + animal	Some synovial/tissue readouts; limited local proof	Mostly no depletion or transfer studies	Strongest and most consistent evidence base
T cells	Crocin/Krocina™; Curcumin; Tanshinone IIA; Cherry seed extract; Curcumin + L.acidophilus+vitamin B	RestoreTh17/Tregbalance; TLR4/MyD88/NF-κB; STAT3 modulation	Clinical + animal + ex vivo	Mainly peripheral blood; limited synovial confirmation	Limited causal proof	Promising translational signal; local validation needed
Neutrophils	CDDO-Me; APPA; RosA-enriched mud-bath therapy; Tetramethylpyrazine	Reduce ROS, NETs, degranulation; Nrf2 activation	*In vitro* + animal + limited clinical	Little synovial evidence	Lacking	Exploratory evidence area
Mast cells	Viscum coloratum extract; N-palmitoyl-D-glucosamine	Inhibit degranulation; FcϵRI-Syk- PLCγ-PKCδ-Akt-MAPK	*In vitro* + animal	Little OA patient validation	Lacking	Relevant to synovitis and pain
B cells	Curcumin (indirect)	BAFF subset modulation	Absent or extremely limited	Absent	Absent	Potential but underdeveloped
DC/NK cells	No direct studies	Unclear; theoretical role	Absent or extremely limited	Absent	Absent	Hypothesis-generating

Immune-cell populations with consistent evidence across several of these dimensions were considered to have a relatively mature evidence base. Evidence based mainly on *in vitro* experiments, animal studies, or peripheral immune-marker changes was regarded as exploratory. When evidence was limited to scattered observations or lacked direct natural-product intervention studies, these cell types were considered potential research directions rather than established therapeutic targets.

## 3 The immune microenvironment in OA: cellular basis for natural-product intervention

Immune dysregulation in OA is not driven by a single immune-cell population. Instead, it reflects continuous interactions among innate immune cells, adaptive immune cells, and the osteochondral unit (15, 20). Understanding the pathological roles of different immune-cell types is therefore essential for evaluating the immunomodulatory effects of natural products in OA.

### 3.1 Innate immune cells in OA pathology

Innate immune cells are involved in local inflammatory responses within osteoarthritic joints (21, 22). Among these cells, macrophages are the most extensively studied and represent a major immune-cell population in natural-product research on OA (23, 24). Macrophage infiltration and activation are frequently observed in OA synovium. Activated macrophages release inflammatory cytokines, chemokines, and matrix-degrading mediators, thereby promoting synovial inflammation, cartilage metabolic imbalance, and pain sensitization (25, 26). Pro-inflammatory macrophage phenotypes are generally associated with exacerbated inflammation and tissue damage (27). In contrast, anti-inflammatory and reparative macrophage phenotypes may limit inflammation and support tissue repair by secreting mediators such as interleukin-10 (IL-10) and transforming growth factor-β (TGF-β) (28, 29).

Neutrophils may contribute to OA pathology depending on disease stage and inflammatory status (30). During active inflammation or early tissue injury, neutrophils can release ROS, proteases, and other inflammatory mediators, thereby amplifying local inflammatory responses (31). Recently, neutrophil extracellular traps (NETs) have been proposed to promote synovial inflammation and cartilage damage, although direct evidence in OA remains limited (32). Compared with inflammatory joint diseases such as RA, neutrophils should not currently be regarded as dominant immune drivers of OA. Rather, they are better considered exploratory immune-cell populations associated with inflammatory amplification.

Mast cells and mast-cell-derived inflammatory mediators have been reported to be increased in the synovial fluid of patients with OA, suggesting a potential association with synovitis development or progression (33, 34). Mast-cell degranulation releases histamine, proteases, prostaglandins, TNF-α, and other mediators. These factors may increase vascular permeability, recruit additional inflammatory cells, and amplify pain responses (35–37). However, the role of mast cells in OA may differ across patients. Current evidence is insufficient to define mast cells as core drivers across all OA cases. Instead, they may be more relevant in patients with prominent synovitis, enhanced mast-cell activation, or heightened pain sensitivity (33, 38, 39). Therefore, mast-cell-associated responses may be relevant to interventions aimed at synovitis and pain sensitization.

Evidence regarding DCs and NK cells in OA remains sparse. In theory, these cells may regulate immune responses through antigen presentation or cytokine secretion. However, current studies have not established a coherent mechanistic framework for their roles in OA. Their abundance, activation status, and causal functions in osteoarthritic joints remain unclear (8, 40, 41). Direct evidence showing that natural products modulate DCs or NK cells in OA is also limited. Thus, DCs and NK cells should currently be regarded as potential research directions rather than established therapeutic targets.

### 3.2 Adaptive immune cells and immune homeostasis in OA

Adaptive immune cells also contribute to immune imbalance in OA, with T cells receiving the greatest attention. Alterations in T-cell subsets have been observed in the synovial fluid, synovium, or peripheral blood of patients with OA (42, 43). Among these alterations, an imbalance between Th17/Treg cells has been associated with persistent inflammation and cartilage degradation. Th17 cells secrete interleukin-17 (IL-17) and other pro-inflammatory mediators that promote inflammation, cartilage catabolism, and extracellular matrix degradation. In contrast, Treg cells suppress excessive inflammatory responses and help maintain immune homeostasis (44, 45).

However, these findings should be interpreted with caution. OA is not a classical autoimmune disease, and the role of T cells in OA should not be directly equated with their role in RA. Existing studies mainly assess T-cell changes in peripheral blood or the spleen. These findings may indicate systemic immune modulation, but they do not directly demonstrate alterations in local joint T-cell responses. Therefore, extrapolating peripheral Th17/Treg changes to local immune remodeling within the joint may overstate their pathological or therapeutic significance.

Compared with T cells, B-cell research in OA remains limited. B-cell infiltration has been observed in the synovium of patients with OA, particularly in those with prominent synovitis. This finding suggests that B cells may participate in inflammation through antigen presentation, cytokine secretion, immunoglobulin production, or complement-related mechanisms (46, 47). Changes in peripheral B-cell subsets also suggest the possible involvement of humoral immunity in certain OA phenotypes (48, 49). However, evidence for local B-cell function, intra-articular antibody responses, and direct regulation by natural products remains insufficient. Therefore, B cells should currently be regarded as potential research directions rather than mature intervention targets.

### 3.3 Immune-cell networks and their interactions with the osteochondral unit

**Figure 1:**
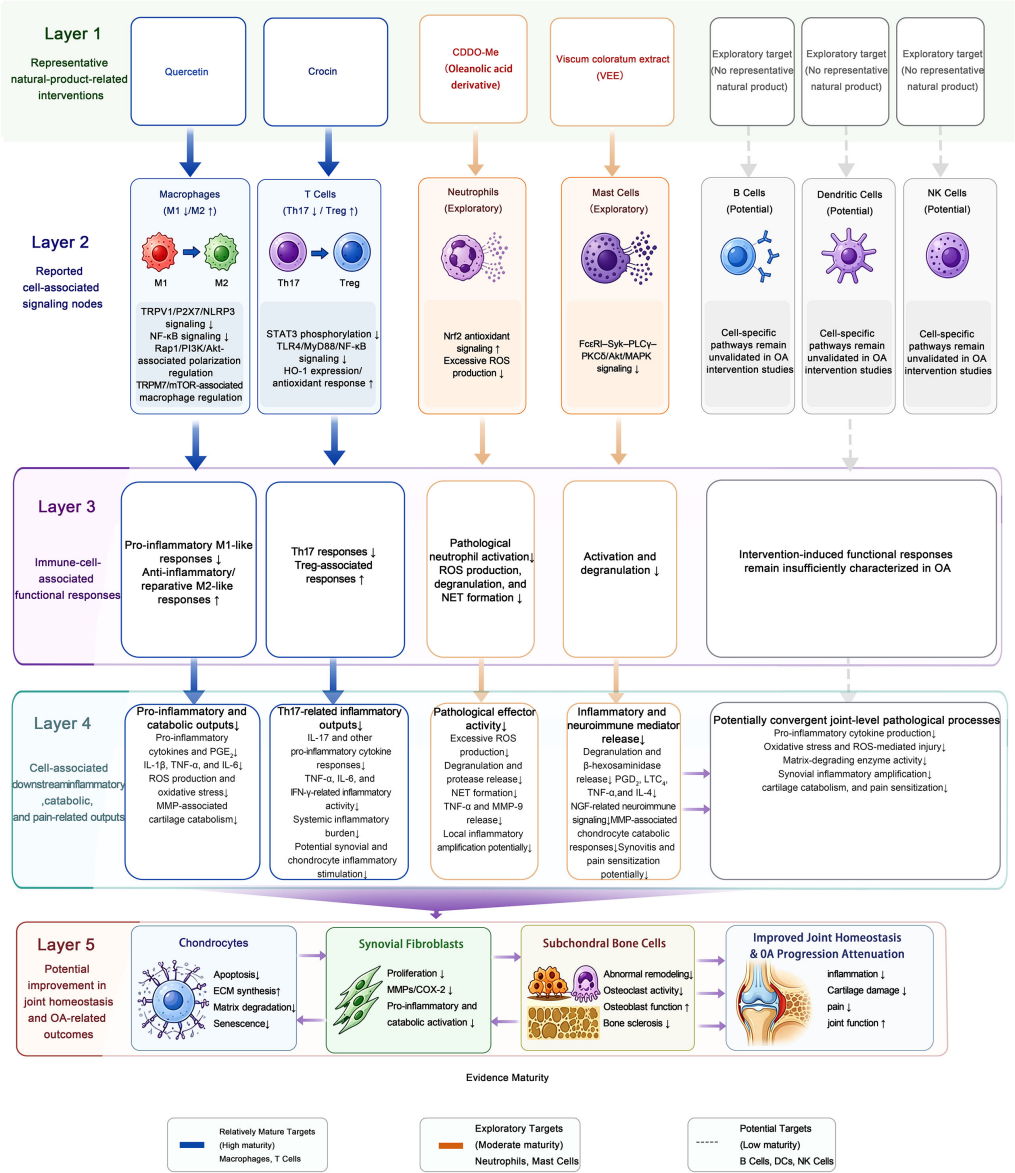
Immune cell-oriented mechanisms and evidence maturity of natural-product interventions in osteoarthritis. *(1) Layer 1 presents one representative intervention for each immune-cell population as an illustrative example and is not intended to provide an exhaustive summary. (2) The listed signaling nodes were reported for one or more representative interventions within each immune-cell category and should not be interpreted as mechanisms shared by all listed natural products. The symbol“↑” indicates an increase, upregulation, promotion, or enhancement, whereas “↓” indicates a decrease, downregulation, inhibition, or reduction.

Immune dysregulation in OA cannot be attributed to a single immune-cell type. Instead, it arises from sustained interactions among innate immune cells, adaptive immune cells, and the osteochondral unit (50). Macrophages and T cells may form inflammatory amplification loops, whereas neutrophils, mast cells, B cells, DCs, and NK cells may participate in local immune responses at specific disease stages or under distinct inflammatory conditions. Inflammatory mediators released by immune cells act on chondrocytes, synovial fibroblasts, and subchondral bone cells. These interactions promote cartilage degradation, synovial hyperplasia, and subchondral bone remodeling. In turn, damaged joint tissues release DAMPs, which further activate immune responses and sustain inflammatory feedback loops (5, 51). This crosstalk between immune cells and joint structural cells is important for understanding how natural products may act in OA. Because of their multi-target properties, natural products may influence several pathological processes simultaneously, including inflammation, oxidative stress, cartilage metabolism, and pain-related signaling. However, without cell-specific experimental evidence, reductions in inflammatory mediators or changes in immune markers remain largely descriptive. These findings do not establish that a specific immune-cell population is a causal therapeutic target. Therefore, future studies should integrate local joint immune profiling with cell-specific functional validation. Ideally, multiple immune-cell populations should be assessed within the same model. This approach may help clarify how natural products remodel the OA immune microenvironment and define the actual contribution of individual immune-cell populations to their protective effects. **Figure 1** illustrates the complex interplay among immune cells and joint structural cells, as well as the potential intervention nodes of natural products in the osteoarthritic microenvironment.

## 4 Evidence for natural-product modulation of OA-associated immune-cell responses

**Figure 2:**
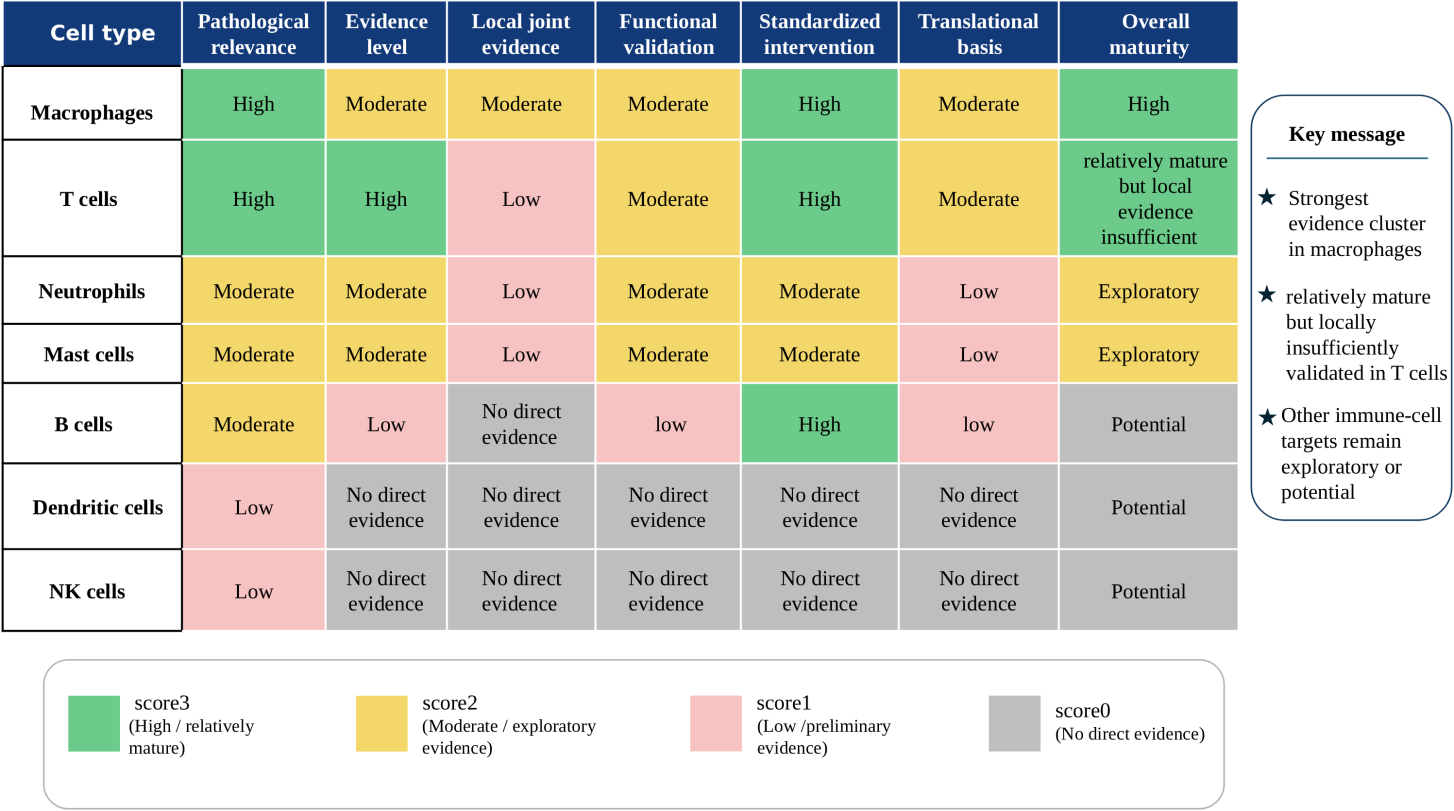
Evidence maturity of natural product modulation of immune cells. *Scores shown in **Figure 2** were assigned according to the criteria defined in **Table 3** and were derived from the evidence-to-score mapping provided in **Table 4**, which links each immune-cell population and scoring dimension to the representative studies discussed in Sections 4.1–4.5.

Natural products may modulate the immune microenvironment in OA by acting on multiple immune-cell populations and immune-related signaling pathways. However, the maturity and strength of evidence vary substantially across immune-cell populations. In the following sections, we summarize representative studies for each immune-cell type and evaluate the corresponding evidence base, with particular attention to mechanistic support, local validation, and translational relevance. To provide a transparent basis for evidence grading,

**Table 2** summarizes representative natural products that modulate immune cells in OA, including their sources, experimental models, regulatory mechanisms, and dosages. **Table 3** defines the maturity scoring criteria used to evaluate evidence related to immune-cell populations across six dimensions. Based on these criteria, **Figure 2** provides a visual summary of the evidence landscape for different immune-cell populations. The key references supporting each score are summarized in **Table 4**, which links each immune-cell population and scoring dimension to the representative studies reviewed in Sections 4.1–4.5. Thus, **Figure 2** should be read together with **Table 3** and **Table 4**, which together summarize the scores, define the scoring thresholds, and provide reference-level traceability. Overall maturity was determined through an integrative qualitative assessment of the six scoring dimensions rather than by simple arithmetic averaging. Accordingly, macrophage-associated responses were considered to have the most mature evidence base because they were supported by the most consistent evidence across pathological relevance, preclinical intervention studies, local tissue-related readouts, and standardized interventions. T cells were considered promising but insufficiently locally validated because the available clinical signals were mainly based on peripheral immune markers. Neutrophil- and mast-cell-associated responses were considered exploratory, because their evidence suggests inflammatory amplification or pain-related mechanisms but remains limited in local and translational validation. B cells, dendritic cells, and NK cells were regarded as potential research directions because direct natural-product intervention evidence, local joint validation, and cell-specific functional support remain scarce or absent.

**Table 2:** Experimental effects and mechanisms of natural products on immune cells in osteoarthritis.

Immune-cell population	Natural product	Main source	Product type	Experimental model	Key markers	Pathway	Dose	Reference
Macrophages	Liquiritin	Glycyrrhiza (Licorice)	Monomer	OA mice	M2 markers↑; TNF-α↓, IL-1β↓, NGF↓, Substance P↓	Rap1/PI3K/ Akt	*In vivo*: 15, 30, 60 mg/ kg; *In vitro*: 25, 50, 100 μM	(53)
	Polydatin	Polygonum cuspidatum	Monomer	OA mice,	M1 markers↓ (iNOS, IL-6, TNF-α); p-STAT1↓, p-STAT3↓; Col2↑, Aggrecan↑	NF-κB/ROS	*In vivo*: 20, 40 mg/kg; *In vitro*: 20, 40 μM	(54)
	THSG	Polygonum multiflorum	Monomer	RAW264.7, OA rats	NO↓, PGE2↓, iNOS↓, COX-2↓, MMP-13↓	–	*In vitro*: 50–400 µg/mL; *In vivo*: 10, 50 mg/kg	(55)
	Quercetin	Various plants	Monomer	OA rats	M1 markers↓ (iNOS, CD86); M2 markers↑ (Arg1, CD163); IL-1β↓, IL-18↓, TNF-α↓	P2X7/NLRP3 pathway	*In vitro*: 3, 8 μM; *In vivo*: 100 mg/kg	(56)
	Apigenin	Vegetables and Herbs (e.g., chamomile)	Monomer	OA mice, RAW264.7cells	M1 markers↓ (IL-1β, IL-6, TNF-α, IL-12); M2 markers↑ (CD206, MG-L1, MG-L2, Arg-1, IL-10)	TRPM7-mTOR	*In vitro*: 10 μM	(57)
	Nystose	Morinda officinalis	Monomer	OA rats, RAW264.7	IL-1β↓, IL-18↓, TNF-α↓, COMP↓, MMP-3↓, Collagen II↑; ROS↓, LDH↓	NLRP3	*In vivo*: 10, 20 mg/kg; *In vitro*: 12.5, 25, 50 μM	(58)
	Caesalpinia sappan	Caesalpinia sappan	Extract	THP-1 macrophages	IL-1β↓, TNF-α↓, NO↓, iNOS↓, COX-2↓	NF-κB	*In vitro*: 5 µg/mL (THP-1)	(59)
	Puerarin	Pueraria (Kudzu root)	Monomer	OA mice, THP-1 macrophages	TNF-α↓, IL-6↓, IL-12↓; TGF-β1↑, IL-10↑	CCL2/CCR2	*In vivo*: 10, 25, 50 mg/ kg; *In vitro*: 25, 50, 100 nM	(60)
T cells	Crocin	Crocus sativus (Saffron)	Monomer	OA patients, PBMCs	Treg↑, Th17↓; IL-17 GMFI↓; Treg/Th17 ratio↑; CRP↓	–	15 mg daily for 4 months	(61)
	Curcumin	Curcuma longa	Monomer	OA patients, PBMCs	Treg↑, Th17↓; Treg/Th17 ratio↑; CD4^+^↓, CD8^+^↓; Total B cells↓, BAFF-R^+^ B cells↓; CRP↓	–	80 mg daily for 3 months	(62)
	Tanshinone IIA	Salvia miltiorrhiza	Monomer	OA rats	Treg cells↑, Th17 cells↓; Foxp3↑, IL-17↓; TNF-α↓, IL-6↓, IL-10↑; aggrecan↑, Collagen II↑	TLR4/MyD88/NF-κB	*In vivo*: 10, 40 mg/kg	(63)
	Sour Cherry Seed Extract	Prunus cerasus	Extract	OA patients, PBMCs	CD4^+^IL-8^+^↓,CD4^+^TNF-α^+^↓, CD4^+^IFN-γ^+^↓,CD4^+^IL-1α^+^↓, CD4^+^IL-1β^+^↓;CRP↓; Leukocyte HO-1↑	–	Topical 5 mL, twice daily for 2 months	(64)
	Curcumin-based combination	Curcuma longa	Combination	OA rats, PBMCs	Th17↓, IL-17↓; Treg↑, IL-10↑; pSTAT3↓; IL-1β↓, TNF-α↓, MMP-13↓; TIMP-3↑	pSTAT3	*In vitro*: 10 μg/mL, In vivo: 500mg/kg	(65)
Neutrophils	CDDO-Me (Bardoxolone methyl)	Oleanolic acid	Semi-synthetic monomer	BM neutrophils, CIOA mice	Nrf2↑; CXCR4^+^ β-Gal^+^↓; TNF-α↓, MMP-9↓; p38 MAPK phosphorylation↓	Nrf2	*In vivo*: 50 μg/knee; *In vitro*: 10–1000 nM	(66)
	APPA	Apocynin +Paeonol	Combination	Human neutrophils	ROS↓; Degranulation (CD63, MPO, elastase)↓; NET formation↓; TNF-α↓, CCL3↓, IL-6↓; Nrf2↑	NF-κB/MAPK	*In vitro*: 10-1000 μg/mL	(67)
	Rosmarinic acid	Salvia rosmarinus	Physical + Phytochemical	OA patients	IL-8↓; IL-6 modestly↑; Cortisol↑; Neutrophil phagocytic activity↑; Microbicidal activity (O_2_^-^ production)↑	–	Topical mud bath (40- 42 °C) with 0.5% Ros A peloid; 10 consecutive days	(68)
	Tetramethylpyrazine	Ligusticum wallichii	Monomer	Network pharmacology	NET-related mechanisms (predicted)	–	–	(69)
Mast cells	Viscum coloratum (VEE)	Viscum coloratum	Extract	RBL-2H3 mast cells, SW1353 chondrocytes	β-hexosaminidase↓, IL-4↓, TNF-α↓, PGD2↓, LTC4↓; MMP-1↓, MMP-3↓, MMP-13↓	FcϵRI/Syk-PLCγ-PKCδ-Akt-MAPK	*In vitro*: IC_50_ 50.59 μg/ mL(TNF-α), 73.28 μg/mL (IL-4), 93.04 μg/ mL (degranulation)	(70)
	N-palmitoyl-D-glucosamine	Synthetic ALIAmide	Monomer	OA rats	Mast cell density↓; CD68^+^ macrophages↓; TNF-α↓, IL-1β↓, NGF↓; MMP-1↓, MMP-3↓, MMP-9↓	–	*In vivo*: 30 mg/kg orally, 3×/week for 21 days	(71)
B cells	Curcumin	Curcuma longa	Monomer	OA patients, PBMCs	Peripheral B cells↓; CD19^+^CD268^+^ (BAFFR)↓; CD19^+^CD267^+^ (TACI)↓; CD19^+^CD269^+^ (BCMA)↓; CD269 GMFI↓; CRP↓	–	80 mg daily for 3 months	(72)

The symbol "↑" indicates an increase, upregulation, promotion, or enhancement, whereas "↓" indicates a decrease, downregulation, inhibition, or reduction.

**Table 3:** Maturity scoring criteria for immune cells in osteoarthritis (linked to **Figure 2**).

Dimension	Score 3 (high/strong)	Score 2 (moderate/intermediate)	Score 1 (low/limited)	Score 0 (none)
Pathological relevance	Clearly identified in osteoarthritis joint tissues, with evidence implicating roles in synovial inflammation, cartilage degradation, pain sensitization, or structural damage.	Detected in OA joint tissues with suggestive but inconsistent evidence for functional involvement.	Limited or indirect evidence of presence, primarily inferred from non-OA disease contexts.	No evidence in OA-relevant tissues.
Evidence level	Supported by randomized controlled trials with complementary animal and/or *in vitro* mechanistic evidence.	Supported by animal and *in vitro* studies with limited or small-scale clinical evidence.	Restricted to *in vitro* studies, single animal models, or computational/network pharmacology predictions.	No direct experimental evidence.
Local joint evidence	Consistently validated in synovial tissue, synovial fluid, cartilage, subchondral bone, or osteochondral units across multiple studies.	Limited but direct evidence from at least one study in joint-resident tissues.	Evidence restricted to peripheral blood, PBMCs, or systemic immune compartments.	No direct OA joint evidence.
Functional validation	Demonstrated through cell depletion, adoptive transfer, or cell-specific genetic manipulation confirming causal involvement in natural-product effects.	Supported by pharmacological inhibition, neutralization, or co-culture approaches without definitive cell-specific causality.	Based on changes in cellular markers, proportions, or signaling activity without causal validation.	No functional evidence.
Standardized intervention	Purified compounds with defined composition, established dosing,	Standardized extracts or defined monomers with known composition	Complex mixtures or multi-component formulations with variable or poorly defined composition.	No defined intervention.
Translational potential	Clinical studies demonstrating improvements in symptoms, function, imaging, structural outcomes, or biomarkers with documented safety.	Clinical evidence limited to symptomatic or functional improvement without structural endpoints.	Evidence restricted to preclinical models or *in vitro* systems.	No translational evidence.

**Table 4:** Evidence-to-score mapping for Immune-cell-associated evidence in **Figure 2**.

Immune-cell population	Scoring dimension	Score	Simplified rationale	Main supporting references
Macrophages	Pathological relevance	3	Strongly implicated in OA synovitis, cartilage degradation, and pain.	(7, 23–29)
Macrophages	Evidence level	2	Supported by multiple *in vitro* and animal studies, but limited clinical evidence.	(53–60)
Macrophages	Local joint evidence	2	Several *in vivo* studies included joint tissue or histopathological assessment, with selected studies reporting macrophage-associated local readouts	(53, 54, 56–58, 60)
Macrophages	Functional validation	2	Pathway and co-culture evidence exists, but causal macrophage-specific validation is limited.	(53–57)
Macrophages	Intervention standardization	3	Most interventions are defined compounds with reported dosing.	(53–60)
Macrophages	Translational potential	2	Preclinical efficacy is consistent, but human and structural evidence is insufficient.	(53–60)
T cells	Pathological relevance	3	Th17/Treg imbalance and IL-17 activity are linked to OA inflammation.	(12, 42–45)
T cells	Evidence level	3	Evidence includes small clinical trials, animal studies, and *in vitro* data.	(61–65)
T cells	Local joint evidence	1	Evidence is mostly peripheral or splenic, with one animal study reporting cartilage IL-17 changes; synovial fluid or synovial tissue T-cell profiling is lacking.	(61–65)
T cells	Functional validation	2	Pathway and cellular evidence exists, but T-cell-specific causal tests are lacking.	(63–65)
T cells	Intervention standardization	3	Several studies used defined or standardized preparations with reported doses.	(61–65)
T cells	Translational potential	2	Clinical studies show immune and symptom improvements, but lack local and structural endpoints.	(61, 62, 64)
Neutrophils	Pathological relevance	2	May amplify OA inflammation, but are not established as dominant drivers.	(11, 30–32)
Neutrophils	Evidence level	2	Evidence includes *in vitro*, animal, and limited human-related findings.	(66–69)
Neutrophils	Local joint evidence	1	Direct evidence in OA synovium or synovial fluid is limited.	(66–69)
Neutrophils	Functional validation	2	ROS, degranulation, NET formation, and phagocytosis have been assessed.	(66–68)
Neutrophils	Intervention standardization	2	Some interventions are defined, but protocols remain heterogeneous.	(66–69)
Neutrophils	Translational potential	1	Evidence remains mainly preclinical and exploratory.	(66–69)
Mast cells	Pathological relevance	2	Associated with synovitis, mediator release, and pain sensitization.	(9, 33–39)
Mast cells	Evidence level	2	Supported by *in vitro* mast-cell systems and limited animal OA studies.	(70, 71)
Mast cells	Local joint evidence	1	Limited animal joint evidence; human OA joint validation is lacking.	(71)
Mast cells	Functional validation	2	Degranulation, mediator release, signaling, and chondrocyte interactions were assessed.	(70, 71)
Mast cells	Intervention standardization	2	Includes a defined fatty-acid amide and a characterized botanical extract.	(70, 71)
Mast cells	Translational potential	1	Evidence remains preclinical despite pain and histological improvements in animals.	(70, 71)
B cells	Pathological relevance	2	B-cell infiltration and humoral activity are reported in selected OA phenotypes.	(13, 46–49)
B cells	Evidence level	1	Peripheral B-cell subset changes were reported as secondary findings after curcumin treatment, without dedicated B-cell-focused mechanistic evidence	(62)
B cells	Local joint evidence	0	No direct local evidence; Ref. 62 provides peripheral evidence only	(62)
B cells	Functional validation	1	Evidence is limited to peripheral B-cell subset changes without causal testing.	(62)
B cells	Intervention standardization	3	Evidence comes from a standardized curcumin preparation.	(62)
B cells	Translational potential	1	Peripheral changes were observed, but no local or structural benefit was shown.	(62)
Dendritic cells	Pathological relevance	1	Possible involvement is suggested, but their role in OA remains unclear.	(41)
Dendritic cells	Evidence level	0	No direct natural-product intervention evidence in OA.	Not available
Dendritic cells	Local joint evidence	0	No local joint evidence for DC modulation by natural products.	Not available
Dendritic cells	Functional validation	0	No DC-specific functional validation is available.	Not available
Dendritic cells	Intervention standardization	0	Not assessable because direct intervention studies are lacking.	Not available
Dendritic cells	Translational potential	0	No translational evidence supports DCs as established intervention targets.	Not available
Natural killer cells	Pathological relevance	1	Possible involvement is suggested, but their OA role remains poorly defined.	(8, 41)
Natural killer cells	Evidence level	0	No direct natural-product intervention evidence in OA.	Not available
Natural killer cells	Local joint evidence	0	No local joint evidence for NK-cell modulation by natural products.	Not available
Natural killer cells	Functional validation	0	No NK-cell-specific functional validation is available.	Not available
Natural killer cells	Intervention standardization	0	Not assessable because direct intervention studies are lacking.	Not available
Natural killer cells	Translational potential	0	No translational evidence supports NK cells as a target.	Not available

### 4.1 Macrophage-associated responses: the most mature evidence base

Macrophage-associated responses currently have the most developed evidence base in OA-related natural-product research. Existing studies mainly focus on macrophage phenotypic regulation, particularly the shift from pro-inflammatory macrophage phenotypes toward anti-inflammatory or reparative phenotypes. In this review, the terms M1-like and M2-like are used as simplified and operational descriptors rather than as a complete representation of macrophage biology in OA. Recent single-cell and high-dimensional immune-profiling studies indicate that macrophages within osteoarthritic joints are highly heterogeneous and cannot be fully classified into two fixed polarization states. Therefore, the M1-like and M2-like phenotypes discussed here mainly refer to marker patterns commonly assessed in natural-product studies. They are not intended to imply a strict binary model of macrophage polarization (52).

The focus on macrophages is biologically plausible because these cells are abundant in osteoarthritic synovium and can regulate synovial inflammation, cartilage matrix degradation, and pain-related signaling. Several natural products have been reported to modulate macrophage phenotypes and macrophage-mediated inflammation in OA models. For example, liquiritin promoted anti-inflammatory M2-like macrophage polarization by inhibiting the Rap1/phosphoinositide 3-kinase (PI3K)/Akt pathway and upregulating M2-associated markers, including CD206, CD163, and arginase-1 (Arg-1) (53). Polydatin suppressed NF-κB signaling, reduced M1-associated marker expression in animal models, and attenuated local inflammation (54). *In vitro*, 2,3,5,4′-tetrahydroxystilbene-2-O-β-D-glucoside (THSG) inhibited inflammatory mediators, including inducible nitric oxide synthase (iNOS), nitric oxide (NO), and prostaglandin E2 (PGE2). THSG also reduced IL-1β-induced matrix metalloproteinase-13 (MMP-13) expression in chondrocytes, suggesting anti-inflammatory and cartilage-protective effects (55). Similarly, quercetin inhibited M1-associated inflammatory factors, including IL-1β, IL-6, and TNF-α, while upregulating M2-associated markers in both *in vitro* experiments and animal models. This effect may be mediated by blockade of the TRPV1/P2X7/NOD-like receptor family pyrin domain containing 3 (NLRP3) signaling axis (56). Apigenin modulated the transient receptor potential melastatin 7 (TRPM7)/mechanistic target of rapamycin (mTOR) pathway in co-culture systems. It suppressed M1-associated cytokines, including IL-1β, IL-6, TNF-α, and interleukin-12 (IL-12), and increased M2-associated markers, including CD206, macrophage galactose-type lectin-1 (MG-L1), macrophage galactose-type lectin-2 (MG-L2), Arg-1, and IL-10. Evidence from animal models further supports its cartilage-protective effects (57). Recent studies also suggest that nystose, Caesalpinia sappan extract, and puerarin may exert protective effects by modulating macrophage-mediated inflammatory responses (58–60).

Overall, macrophage-related studies provide a relatively coherent body of evidence. Current evidence includes *in vitro* experiments and animal studies assessing histological changes, inflammatory mediators, and macrophage-associated phenotypic markers. Most findings suggest that natural products can attenuate synovial inflammation, inhibit cartilage matrix degradation, and, in some models, reduce pain sensitivity or improve joint function by regulating M1-like/M2-like macrophage-associated responses. Although natural products associated with macrophage modulation act through different upstream signaling events, their effects appear to converge on a shared pattern of macrophage regulation. Most interventions reduce pro-inflammatory macrophage activation by inhibiting NF-κB- or NLRP3-related signaling, attenuating oxidative stress, and, in some cases, promoting PI3K/Akt-associated M2-like polarization. For example, liquiritin has been associated with the Rap1/PI3K/Akt axis, quercetin with the TRPV1/P2X7/NLRP3 pathway, apigenin with TRPM7/mTOR-related signaling, and polydatin with inhibition of NF-κB/ROS-associated inflammatory responses. These compounds generally reduce M1-associated mediators, such as IL-1β, TNF-α, and IL-6, while increasing M2-associated markers, including CD206, Arg-1, and IL-10. Thus, the available evidence suggests that modulation of macrophage phenotypes may represent a common downstream feature of these natural products and may contribute to the attenuation of OA-associated synovial inflammation.

However, several evidence gaps remain, which can be grouped into four interrelated issues. First, macrophage states within OA joints are highly heterogeneous, and the conventional M1/M2 framework cannot fully capture their functional diversity. Although this framework remains useful for organizing existing natural-product studies, future work should move beyond a limited set of polarization markers and incorporate single-cell profiling, spatial analysis, and functional validation to define the macrophage subsets that are truly affected by natural products. Second, direct comparative studies among representative compounds are currently lacking. It remains unclear whether the simultaneous modulation of multiple macrophage-regulatory pathways would produce additive or synergistic effects. Third, most existing studies infer macrophage involvement from changes in macrophage-associated markers and inflammatory mediators. Because macrophage depletion, adoptive transfer, macrophage-specific pathway blockade, and cell-specific analyses have rarely been incorporated into experimental designs, the causal contribution of macrophages to the protective effects of natural products remains insufficiently established. Finally, natural products usually exert multi-target effects and may simultaneously affect chondrocytes, synovial fibroblasts, oxidative stress, and pain-related pathways. Therefore, their overall protective effects should not be attributed solely to macrophage regulation. Future studies should integrate local joint immune profiling, direct compound comparisons, and macrophage-specific functional validation to clarify how macrophage-associated responses contribute to natural-product-mediated protection in OA and to determine whether these responses translate into clinically meaningful structural or symptomatic benefits.

### 4.2 T-cell-associated responses: clinically relevant signals but insufficient local validation

Among adaptive immune-cell populations, T cells have received the greatest attention in natural-product research on OA. Current studies mainly focus on the balance between Th17 and Treg cells. Compared with macrophage-focused research, T-cell evidence more often derives from analyses of peripheral blood, splenic immune-cell populations, or systemic inflammatory markers. These findings suggest immunomodulatory effects, but their relevance to local joint pathology should be interpreted cautiously. Several clinical studies have reported T-cell-related immune changes after natural-product-based interventions. In a randomized clinical trial, Krocina™ increased Treg-cell frequency and reduced Th17-cell frequency in the peripheral blood of patients with OA. It also decreased the geometric mean fluorescence intensity of interleukin-17 (IL-17), shifted the Treg/Th17 ratio toward Treg predominance, and reduced serum C-reactive protein (CRP) levels. These findings support systemic immune modulation by the crocin-based intervention (61). Similarly, oral curcumin (Sinacurcumin^®^), administered for three months, increased peripheral Treg-cell frequency, decreased Th17-cell frequency, shifted the Treg/Th17 ratio, and reduced the proportions of CD4^+^ and CD8^+^ T cells (62). Preclinical studies further support the potential involvement of T cells in natural-product-mediated modulation of OA. In rats with OA, tanshinone IIA regulated the Th17/Treg balance, reduced TNF-α and IL-6 levels, and decreased IL-17 expression in cartilage. These effects may involve the Toll-like receptor 4 (TLR4)/myeloid differentiation primary response 88 (MyD88)/NF-κB pathway (63). In a small clinical study, cherry seed extract upregulated heme oxygenase-1 (HO-1) and reduced interleukin-8 (IL-8), TNF-α, interferon-γ (IFN-γ), interleukin-1α (IL-1α), and interleukin-1β (IL-1β) production by activated CD4^+^ T cells. These immunological changes were correlated with improvements in visual analog scale (VAS) pain scores and reductions in CRP levels (64). Combination interventions have also provided evidence of T-cell-related modulation. For example, a combination of curcumin, Lactobacillus acidophilus, and B vitamins alleviated pain and cartilage damage in a monoiodoacetate (MIA)-induced rat model. *In vitro* experiments using peripheral blood mononuclear cells (PBMCs) suggested that this combination may influence the Th17/Treg balance by inhibiting signal transducer and activator of transcription 3 (STAT3) phosphorylation (65).

Overall, T-cell-related studies provide a relatively limited but clinically relevant line of evidence. A recurring signal across existing intervention studies is the partial restoration of the Th17/Treg balance and attenuation of IL-17-related inflammatory activity. Crocin/saffron-derived interventions and curcumin have been associated with increased Treg-cell frequency, reduced Th17-cell responses, improved Treg/Th17 ratios, and lower systemic inflammatory markers in patients with OA. In experimental models, tanshinone IIA appears to regulate the related immune axis through TLR4/MyD88/NF-κB signaling, whereas the combination of curcumin, Lactobacillus acidophilus, and B vitamins mainly involves STAT3 signaling. By contrast, cherry seed extract shows a somewhat different profile, mainly reducing pro-inflammatory cytokine production by activated CD4^+^ T cells and lowering systemic inflammatory burden. Collectively, these findings suggest that natural products targeting T-cell-related immune responses may not share a single upstream mechanism. Instead, their effects appear to converge on partial restoration of T-cell immune homeostasis. However, the current evidence should be interpreted cautiously. Unlike macrophage-focused studies, which often include local synovial or joint tissue readouts in animal models, most T-cell studies rely mainly on peripheral blood or circulating T-cell subsets. Therefore, these findings are better interpreted as evidence of systemic immune modulation rather than direct evidence of intra-articular T-cell remodeling.

It remains unclear whether changes in the peripheral Th17/Treg balance reflect a causal role in local immune regulation or represent downstream markers of reduced systemic inflammation. This uncertainty is reinforced by the limited use of T-cell depletion, adoptive transfer, and T-cell-specific pathway blockade in experimental studies. In addition, clinical studies rarely include structural endpoints, such as magnetic resonance imaging (MRI)-assessed synovitis, cartilage thickness, or subchondral bone changes. It is therefore difficult to determine whether T-cell modulation contributes to disease modification rather than short-term improvement in inflammatory activity or symptoms. Future studies should integrate synovial or synovial-fluid T-cell profiling, functional validation experiments, and phenotype-stratified clinical trials with structural outcomes.

### 4.3 Neutrophil-associated responses:exploratory evidence for inflammatory amplification

Research on neutrophils in OA remains at an exploratory stage. Compared with macrophage- and T-cell-focused studies, neutrophil-related evidence is limited and heterogeneous in terms of experimental models and intervention types. Current studies mainly focus on abnormal neutrophil activation, oxidative stress, degranulation, neutrophil extracellular trap (NET) formation, and neutrophil senescence. CDDO-Me, a semi-synthetic triterpenoid derived from oleanolic acid and an activator of nuclear factor erythroid 2–related factor 2 (Nrf2), has been reported to modulate neutrophil inflammatory status in an OA model. It upregulated Nrf2 expression, reduced the proportion of senescence-like neutrophils, and decreased inflammatory and tissue-injury markers, including TNF-α and matrix metalloproteinase-9 (MMP-9) (66). APPA, a combination of apocynin and paeonol, inhibited neutrophil degranulation, ROS production, and NET formation in an *in vitro* human neutrophil model. Importantly, APPA largely preserved phagocytic and antimicrobial functions, suggesting that it may suppress pathological neutrophil activation without broadly impairing host-defense capacity (67). Rosmarinic-acid-enriched mud-bath therapy has also been reported to reduce peripheral IL-8 levels in patients with OA and to enhance neutrophil phagocytosis and oxygen-dependent bactericidal activity (68). In addition, tetramethylpyrazine has been proposed to exert therapeutic effects in OA through mechanisms related to NET formation; however, this conclusion is based on network pharmacology predictions and still requires experimental validation (69).

These findings suggest that natural products may reduce tissue injury by limiting excessive neutrophil-driven inflammatory responses. Some interventions appear to preserve essential neutrophil functions while suppressing pathological activation. This feature distinguishes these interventions from broad-spectrum anti-inflammatory strategies and supports the possibility that neutrophil-associated responses may represent auxiliary or exploratory mechanisms in OA immunomodulation. Overall, neutrophil-related evidence remains preliminary, but it provides mechanistic clues for understanding inflammatory amplification in OA. Unlike macrophages and T cells, neutrophils have not been established as major drivers of OA progression. However, under specific pathological conditions, they may participate in the amplification of local inflammation. The limited intervention studies available to date suggest a shared pattern: natural products may attenuate excessive neutrophil activation by reducing ROS production, degranulation, NET formation, and pro-inflammatory mediator release. Nevertheless, different interventions appear to act through distinct mechanistic nodes. CDDO-Me seems to reduce senescence-like neutrophils mainly through Nrf2-related antioxidant activity, whereas APPA more directly inhibits pathological neutrophil activation, including degranulation, ROS production, and NET formation, while preserving antimicrobial function. Rosmarinic-acid-enriched mud-bath therapy has been associated with modulation of peripheral neutrophil function, whereas the proposed association between tetramethylpyrazine and NET formation remains hypothesis-generating and requires experimental confirmation. Despite these differences, these interventions share a common implication: they may selectively limit harmful neutrophil responses without broadly suppressing innate immune function. However, the current evidence remains insufficient. Most studies have assessed systemic or experimentally induced neutrophil activation markers, whereas direct evidence from the OA synovium, synovial fluid, or local neutrophil microenvironment is still lacking. It also remains unclear whether reduced neutrophil activation directly contributes to cartilage protection, pain relief, or long-term structural improvement, or whether it simply reflects a broader reduction in inflammatory burden. Future studies should combine local joint neutrophil profiling with functional assays of NET formation, degranulation, and oxidative activity, together with structural and pain-related endpoints. Such studies will help determine whether neutrophils can be considered therapeutically relevant targets in specific OA phenotypes, particularly those characterized by active synovitis or inflammatory flares.

### 4.4 Mast-cell-associated responses: exploratory evidence for synovitis and pain sensitization

Studies on mast cells in OA remain limited. The association of mast cells with synovial inflammation and pain sensitization suggests that mast-cell-associated responses may represent exploratory mechanisms through which natural products exert protective effects in selected OA phenotypes. Existing natural-product studies mainly focus on mast-cell activation, degranulation, and the downstream release of inflammatory mediators. Some studies have also examined whether these changes affect chondrocyte injury and pain-related outcomes. For example, Viscum coloratum ethanol extract (VEE) inhibited β-hexosaminidase release and reduced the production of multiple inflammatory mediators in immunoglobulin E (IgE)-/antigen (Ag)-activated RBL-2H3 cells. VEE also downregulated the high-affinity IgE receptor (FcϵRI) and its downstream Syk/phospholipase Cγ (PLCγ)/protein kinase Cδ (PKCδ)/Akt/mitogen-activated protein kinase (MAPK) signaling axis. In a model in which chondrocytes were stimulated with mast-cell-derived inflammatory mediators, VEE reduced the expression and activity of matrix metalloproteinase-1 (MMP-1), matrix metalloproteinase-3 (MMP-3), and matrix metalloproteinase-13 (MMP-13). These findings suggest that VEE may attenuate cartilage catabolism by inhibiting mast-cell-derived inflammatory signals (70). Autacoid local injury antagonism amides (ALIAmides) are naturally occurring fatty acid amides. N-palmitoyl-D-glucosamine (PGA) is a representative compound in this class. *In vivo*, PGA reduced mast-cell density in a MIA-induced rat model of OA. PGA also decreased TNF-α, IL-1β, nerve growth factor (NGF), and MMP levels. These changes were accompanied by reduced mechanical allodynia and improved joint histopathology (71).

Overall, mast-cell-related evidence remains limited, but it provides a useful neuroimmune perspective for natural-product research in OA. Unlike macrophage-focused studies, which mainly emphasize inflammatory polarization and cartilage catabolism, mast-cell studies more often focus on degranulation, mediator release, synovial inflammation, and pain sensitization. VEE has been reported to inhibit IgE/Ag-induced mast-cell activation and reduce mast-cell-derived mediators, including β-hexosaminidase, interleukin-4 (IL-4), TNF-α, prostaglandin D2 (PGD2), and leukotriene C4 (LTC4). These effects were accompanied by reduced expression of matrix-degrading enzymes in chondrocytes, suggesting a possible mast-cell–chondrocyte interaction. PGA provides another line of preclinical evidence. Its ability to reduce mast-cell density, NGF, inflammatory mediators, and mechanical allodynia in an OA model suggests that mast-cell regulation may be linked to pain-related outcomes.

Taken together, these findings suggest that natural products targeting mast-cell-related responses may be more relevant to OA phenotypes characterized by synovitis, neurovascular activation, or pain sensitization. However, this interpretation remains preliminary. Current evidence is mainly derived from *in vitro* mast-cell activation systems and animal models, whereas direct validation in human OA synovium or synovial fluid remains limited. It is also unclear whether the observed effects result from direct actions on mast cells or from broader anti-inflammatory or chondroprotective effects of the tested natural products. Another unresolved question is whether changes in mast-cell activity represent an independent therapeutic mechanism or a secondary consequence of reduced synovial inflammation. These uncertainties point to a central evidence gap: direct evidence linking mast-cell functional status in local joint tissues to the effects of natural-product interventions remains insufficient. Future studies should integrate local mast-cell profiling, degranulation assessment, measurement of mast-cell-derived mediators, analysis of neuroimmune pathways such as NGF signaling, and pain-related endpoints. Such studies may help determine whether mast-cell-related pathways are therapeutically relevant in synovitis-dominant or pain-sensitive OA phenotypes.

### 4.5 B cells, dendritic cells, and NK cells: potential research directions with limited direct evidence

Compared with macrophages, T cells, neutrophils, and mast cells, evidence for B cells, DCs, and NK cells in OA-related natural-product research remains substantially less mature. At present, these cell populations should not be regarded as independent therapeutic targets. They may participate in the OA immune microenvironment through antigen presentation, antibody-related responses, cytokine production, innate immune surveillance, and interactions with synovial stromal cells. However, direct evidence linking natural-product interventions to functional regulation of these cells in OA remains limited. Among these three cell populations, B cells have only limited evidence from secondary findings in individual clinical studies. For example, in addition to regulating the Th17/Treg balance, curcumin was reported to reduce the frequency of total peripheral B cells and certain B-cell activating factor receptor-related subsets in patients with OA (62). However, this finding should be interpreted as indirect evidence. No study has yet determined whether natural products affect B-cell infiltration, local antibody production, or complement activation in OA synovial tissue or synovial fluid. Therefore, it remains unclear whether the observed B-cell changes represent a direct effect of natural products or an accompanying phenomenon associated with broader reductions in inflammation. The evidence for DCs and NK cells is even more preliminary. Although both cell types have important immunoregulatory functions, current natural-product intervention studies in OA have not systematically assessed their abundance, maturation status, activation phenotype, or functional activity. Scattered theoretical considerations are not sufficient to establish a substantive evidence base. Therefore, no definitive mechanistic conclusions can currently be drawn for these cell populations in the context of natural-product-based OA interventions. They should be positioned as hypothesis-generating research directions rather than validated immune targets. This evidence gap is important. For B cells, DCs, and NK cells, OA-related natural-product research lacks direct assessment in local joint tissues, cell-specific functional validation, and a causal chain linking intervention-induced immune changes to disease-relevant outcomes. To move these hypothetical targets toward testable therapeutic relevance, future studies should strengthen several aspects. First, single-cell or spatial transcriptomic analyses should be performed in OA synovium or synovial fluid to define the presence, activation state, and spatial organization of these cell populations. Second, natural-product intervention studies should include local tissue or synovial fluid sampling for immune phenotyping whenever feasible. Third, where appropriate, cell depletion, adoptive transfer, or pathway-specific perturbation should be used to test their causal contribution.

Across immune-cell populations, the available evidence indicates different degrees of support for natural-product-mediated immunomodulation in OA. Studies involving macrophage- and T-cell-associated immune responses currently provide the most consistent evidence, mainly involving macrophage phenotypic regulation and partial restoration of the Th17/Treg immune balance. Neutrophils and mast cells appear to be more context-dependent targets, particularly in relation to inflammatory amplification, synovitis, neuroimmune activation, and pain sensitization. In contrast, B cells, DCs, and NK cells remain largely exploratory because direct evidence from natural-product intervention studies is still limited. Across these immune-cell populations, many natural products exert broad anti-inflammatory effects, including reduced inflammatory mediator production, attenuation of oxidative stress, and modulation of immune-cell activation states. However, their cell-specific actions, causal contribution to joint protection, and relevance to distinct OA phenotypes remain insufficiently defined. Future studies should integrate local joint immune profiling, cell-specific functional validation, direct comparison of representative compounds, and phenotype-stratified clinical trials to determine which immune-cell-associated responses are most likely to mediate clinically meaningful responses to natural-product interventions.

## 5 Translational limitations and future directions

Although current evidence suggests that natural products may modulate immune and inflammatory processes in OA, it remains insufficient to demonstrate clinically meaningful benefits or disease-modifying effects. The principal limitations involve three closely related issues: predominantly preclinical or peripheral-marker-based evidence, insufficient cell-specific functional validation, and heterogeneous, insufficiently characterized natural-product interventions. Most studies have been conducted using *in vitro* experiments or animal models, whereas clinical investigations are often limited by small sample sizes, short follow-up periods, and an emphasis on symptom-based outcomes. Direct evidence demonstrating that natural-product interventions alter the local immune microenvironment of osteoarthritic joints remains scarce. Moreover, although changes in immune-cell proportions, phenotypic markers, or inflammatory mediators are frequently observed alongside improvements in cartilage integrity or other joint-related outcomes, these parallel changes do not establish that a specific immune-cell population is required for, or directly mediates, the observed protective effects. Accordingly, immune modulation should currently be interpreted as a plausible component of the broader pharmacological effects of natural products, rather than as definitive evidence of immune-cell-directed disease modification. Addressing these limitations will require more robust evidence from local joint tissues, cell-specific functional validation, standardized interventions, and phenotype-informed clinical studies.

### 5.1 Limitations in evidence type and functional validation

Most studies of natural-product interventions in OA rely heavily on *in vitro* experiments and animal models. Their conclusions are commonly based on changes in immune-cell numbers, relative proportions, phenotypic markers, or inflammatory mediators. Although these observations are informative, they are primarily descriptive and do not establish whether a particular immune-cell population is necessary for, or directly mediates, the observed protective effects.

This limitation is especially relevant when mechanistic interpretations are based on peripheral immune markers. Changes in circulating immune cells or cytokines may indicate systemic immunomodulation, but they do not necessarily reflect immune changes in the synovium, synovial fluid, or osteochondral unit (72, 73). Because OA pathology is centered in the affected joint, conclusions drawn solely from peripheral blood may overestimate the extent to which natural products remodel intra-articular immune responses. Peripheral immune changes should therefore be interpreted as supportive or surrogate evidence unless they are linked to local joint findings. Paired assessment of peripheral and local immune markers would help determine whether systemic changes correspond to immune alterations within the affected joint.

Similarly, reductions in pro-inflammatory mediators or increases in anti-inflammatory markers support the modulation of immune activity, but they do not demonstrate that the corresponding immune cells or pathways are indispensable for cartilage protection, reduced synovitis, pain relief, or other disease-related outcomes. In many studies, immune-marker changes occur in parallel with broader effects on chondrocytes, synovial fibroblasts, oxidative stress, matrix metabolism, or pain-related signaling. This overlap complicates efforts to determine whether immune regulation is the primary mechanism, one component of a broader multi-target response, or a secondary consequence of reduced tissue injury. Stronger causal evidence will require approaches such as cell depletion, adoptive transfer, cell-specific pathway inhibition, lineage-restricted genetic manipulation, or functional analyses performed in defined immune-cell populations. In OA, these approaches should be combined with local joint immune profiling and disease-relevant outcomes whenever feasible, because systemic immune-cell manipulation may not fully capture tissue-specific effects in the synovium, synovial fluid, or osteochondral unit. Such designs may help establish a mechanistic sequence linking natural-product exposure to immune-cell alteration and, subsequently, to tissue-level or clinical benefit. In the absence of such validation, changes in immune markers should be regarded as mechanistic clues rather than definitive evidence that a specific immune-cell population is the principal target of a natural-product intervention (74, 75).

### 5.2 Intervention heterogeneity and insufficient clinical validation

In addition to the limitations of current mechanistic evidence, substantial heterogeneity among natural-product interventions further restricts cross-study comparability and clinical translation. Current studies include purified compounds, standardized extracts, complex formulations, and combination therapies. These interventions differ in chemical composition, dose, route of administration, formulation characteristics, bioavailability, and batch consistency. As a result, apparently similar interventions may not produce comparable systemic or intra-articular exposures, even when evaluated in the same disease model.

For mechanistic studies, purified compounds and well-characterized standardized extracts offer clear advantages because their composition and biological effects can be more readily defined. By contrast, complex formulations and combination therapies may show therapeutic activity, but their multicomponent nature makes it difficult to identify the principal active constituents, determine dose–response relationships, and attribute observed immune effects to a specific component or immune-cell population. This problem is compounded by incomplete reporting of product composition, extraction procedures, purity, dose selection, formulation characteristics, and batch-to-batch consistency. Without adequate chemical characterization and exposure assessment, it is difficult to determine whether between-study differences reflect true biological variation or differences in the interventions themselves.

Many preclinical studies also lack pharmacokinetic analyses, tissue-distribution data, dose–response assessments, and long-term evaluations. Findings derived solely from *in vitro* systems or short-term animal models should therefore be regarded as early-stage evidence, particularly when the tested concentrations are not linked to systemically or locally achievable exposures. Biological activity under experimental conditions does not establish that therapeutically effective concentrations can be achieved safely in patients or maintained within osteoarthritic joints. This limitation is especially relevant for compounds with low bioavailability, rapid metabolism, or uncertain joint penetration.

Clinical evidence remains limited. Existing studies generally involve small cohorts, short intervention periods, and heterogeneous treatment protocols. Outcomes are often restricted to pain scores, functional measures, circulating cytokines, or peripheral immune-cell phenotypes (61, 62). Although these endpoints may indicate symptomatic improvement or systemic immunomodulation, they do not establish that the intervention alters local joint pathology or slows structural disease progression. This distinction is particularly important for peripheral immune findings, such as changes in the circulating Th17/Treg balance or serum inflammatory mediators. Such changes may be associated with a reduced systemic inflammatory burden, but it remains unclear whether they correspond to changes in synovitis, cartilage loss, subchondral bone abnormalities, or other structural outcomes in OA. The extent to which peripheral immune changes reflect intra-articular responses therefore remains uncertain. Accordingly, improvements in peripheral immune markers and symptom scores should not be interpreted as evidence of disease modification in the absence of local or structural validation. Future clinical studies should therefore combine symptom and functional outcomes with local joint, imaging, and structural endpoints. Relevant measures may include MRI-assessed synovitis, cartilage morphology or thickness, bone marrow lesions, osteophyte progression, joint-space narrowing, synovial-fluid immune profiling, and, where feasible, synovial biopsy-based analyses. Longitudinal sampling is also needed to determine whether immune-marker changes precede clinical improvement, occur in parallel with it, or arise as secondary consequences of reduced disease activity. OA pain is influenced by synovitis, mechanical loading, subchondral bone changes, neural sensitization, and psychosocial factors (76). Pain reduction alone therefore cannot be used to infer immune-cell-specific activity or structural benefit. Similarly, short-term functional improvement does not necessarily indicate modification of the underlying joint pathology. Future trials should integrate immune-related changes with local pathology, imaging progression, pain phenotypes, physical function, and relevant biomarkers. These outcomes should be selected according to the proposed mechanism of action rather than applied as a uniform endpoint set across all OA phenotypes. Such designs will help determine whether natural-product interventions provide benefits beyond short-term symptom relief and whether the observed immune effects are clinically and structurally meaningful.

### 5.3 Future directions: local mechanistic resolution, intervention standardization, and phenotype- and endotype-informed clinical validation

Future translation requires progression from local mechanistic characterization and cell-specific causal validation, through intervention standardization, to phenotype- and endotype-informed clinical testing. The first priority is mechanistic resolution within the affected joint. Future studies should incorporate paired analyses of peripheral blood and synovial fluid or synovial tissue to determine whether systemic immune changes correspond to intra-articular immune remodeling. Single-cell sequencing, spatial transcriptomics, and cell-specific functional analyses may then be used to identify disease-relevant immune-cell states, define their spatial relationships with stromal, vascular, cartilaginous, and bone compartments, and determine whether changes in cell abundance are accompanied by changes in cellular function. Local metabolic, lipidomic, and immunometabolic analyses may provide complementary information on the interactions among immune activation, adipokine-related disturbances, tissue metabolism, and immune–bone crosstalk within the joint microenvironment (77).

These molecular profiles should be linked to functional perturbation and disease-relevant outcomes rather than interpreted as descriptive endpoints alone. Cell depletion, adoptive transfer, lineage-restricted manipulation, or cell-specific pathway inhibition should be incorporated where experimentally feasible to determine whether a candidate immune-cell population is necessary for, or materially contributes to, the protective effects of a natural product. Corresponding outcomes should include synovitis, cartilage integrity, subchondral bone remodeling, pain-related behavior, and physical function. For macrophages and T cells, which currently have relatively stronger supporting evidence, the priority is to quantify their actual contribution to the protective effects associated with natural-product interventions. For neutrophils and mast cells, studies should determine whether their involvement is restricted to particular inflammatory, synovitis-associated, or pain-related states. For B cells, DCs, and NK cells, local abundance, activation status, and functional relevance should first be established before these populations are considered independent therapeutic targets (78).

The second priority is intervention standardization. Future studies should define the chemical composition, active constituents, purity, dose, formulation, route of administration, and batch consistency of natural-product interventions (79). These data should be integrated with pharmacokinetic, bioavailability, and tissue-distribution analyses to determine whether biologically active concentrations are achieved systemically and within the joint. For complex extracts and combination formulations, analytical characterization should be sufficient to distinguish true biological variation from variability arising from product composition or exposure. Only standardized and adequately characterized interventions can support reproducible mechanistic comparisons and credible clinical translation. The third priority is phenotype- and endotype-informed clinical validation. Clinical phenotypes describe observable patterns of disease, whereas endotypes refer to biologically defined mechanisms that may underlie those patterns. Patient selection should therefore integrate clinical presentation, imaging features, local immune biomarkers, systemic inflammatory or metabolic markers, and assessments of pain-related mechanisms. Stratification variables and mechanistic hypotheses should be specified prospectively rather than derived only from *post hoc* subgroup analyses (80, 81).

The phenotype–target–intervention relationships outlined below should be regarded as hypotheses for stratified testing rather than as criteria for clinical treatment selection. Evidence that quercetin and apigenin modulate macrophage-associated inflammatory responses provides a rationale for evaluating these compounds in patients with imaging-defined synovitis and biomarker-supported macrophage-associated inflammatory activity. Such studies should include local macrophage profiling, synovitis-sensitive imaging, and structural and symptomatic outcomes. Experimental evidence that *Viscum coloratum* ethanol extract inhibits mast-cell activation and degranulation similarly supports its investigation in synovitis- or pain-associated subgroups in which local mast-cell activation is objectively demonstrated rather than inferred from symptoms alone. For patients with metabolic syndrome-associated OA or low-grade systemic inflammation, curcumin, saffron-derived compounds, and crocin represent candidates for testing within a metabolically and immunologically defined endotype. The rationale derives from preliminary evidence of changes in peripheral Th17/Treg balance, systemic inflammatory burden, CRP, TNF-α, and selected metabolic indicators rather than from established preferential efficacy in this subgroup (82). Trials should therefore combine metabolic measures with systemic and local immune markers and joint-specific clinical or structural outcomes. For pain-sensitive phenotypes, the involvement of mast cells and mast-cell-derived mediators, including nerve growth factor and histamine, provides a rationale for evaluating N-palmitoyl-D-glucosamine in patients with evidence of neuroimmune or mast-cell-associated pain amplification. Because OA pain is multifactorial, such studies should evaluate synovitis, subchondral bone abnormalities, neural sensitization, and relevant psychosocial contributors in parallel; symptomatic improvement alone should not be interpreted as evidence of mast-cell-specific activity. Mechanical overload-dominant OA represents a distinct clinical phenotype in which immune inflammation may be secondary rather than the principal disease driver. In this setting, immunomodulatory natural products should be tested as adjunctive approaches to attenuate secondary inflammation or oxidative stress, while biomechanical correction, weight management, rehabilitation, and load-modifying strategies remain central. This negative stratification is important because a phenotype- and endotype-informed framework should identify not only patients in whom immune-modulating interventions warrant testing, but also those in whom immune targeting is unlikely to address the pathogenic mechanism.

A rigorous translational trial should therefore connect four elements: a clearly defined clinical phenotype or biological endotype, a plausible and measurable immune-cell-associated mechanism, a standardized natural-product intervention, and mechanistically aligned local immune, imaging, structural, symptomatic, and functional endpoints. Longitudinal sampling should determine whether immune changes precede, accompany, or follow clinical and structural responses. Overall, natural products remain promising candidates for OA immunomodulation. Recent reviews have summarized natural herbs, traditional Chinese medicine formulations, and natural products in OA, including knee OA, with emphasis on inflammation, oxidative stress, autophagy, cartilage metabolism, and signaling pathways such as NF-κB, AMP-activated protein kinase (AMPK), PI3K/Akt, TGF-β/Smad, and Wnt/β-catenin (83–85). The present review complements these compound- and pathway-centered perspectives by evaluating the maturity of evidence across individual immune-cell populations. The next step is not simply to identify additional compounds that alter immune markers, but to determine whether standardized interventions produce reproducible and functionally relevant immune changes in biologically defined patient subgroups. The evidence maturity framework previously presented in **Table 3** and **Table 4** and **Figure 2** may provide a structured approach for assessing progress from descriptive immune-marker changes toward locally validated, functionally supported, and clinically relevant immunomodulatory evidence. Together, local immune evidence, intervention standardization, patient stratification, and mechanistically aligned endpoints will be essential for evaluating translational relevance. **Table 5** summarizes the principal limitations and corresponding research priorities.

**Table 5:** Limitations and future directions of natural product-based immune interventions in osteoarthritis.

Limitation	Typical manifestation	Why it matters	Recommended improvement	Suggested outcome measures
Evidence dominated by *in vitro*/animal models	Few human studies	Poor translation	Conduct multicenter clinical trials	Symptoms; imaging; biomarkers
Peripheral immune readouts overused	Blood Treg/Th17 or cytokines only	May not reflect joint immunity	Collect synovium, synovial fluid, osteochondral samples	Local immune-cell states; histology; omics
Insufficient causal validation	Marker changes only	Cannot confirm the responsible cell populations	Use depletion, adoptive transfer, pathway blocking, cell-specific assays	Causal contribution
Heterogeneous interventions	Monomers, extracts, formulas, combinations	Hard to compare mechanisms	Standardize ingredient, purity, dose, route, batch consistency	Reproducible efficacy; PK data
Short follow-up/symptom-heavy endpoints	Pain relief emphasized	Not equal to disease modification	Include MRI/ultrasound synovitis, cartilage thickness, subchondral bone	Structural outcomes
OA heterogeneity ignored	All patients treated as one group	Effect dilution	Use phenotype/endotype-based stratification	Better responder identification
Limited high-resolution immune profiling	Coarse M1/M2 or Th17/Treg categories	Overlooks heterogeneity	Adopt single-cell, spatial transcriptomics, multiplex imaging	Refined mechanism discovery

## 6 Conclusion

Current evidence indicates that natural products can modulate immune and inflammatory activity associated with OA. However, given the pleiotropic pharmacological properties of natural products, immune-cell changes observed in many studies may represent only one component of broader protective responses. These responses may also involve joint structural cells, inflammatory mediators, oxidative stress, and pain-related pathways. Therefore, it often remains difficult to distinguish direct regulation of specific immune-cell populations from non-specific immune changes secondary to reduced inflammation. Immune-marker alterations should therefore be interpreted as mechanistic clues rather than definitive evidence of immune-cell-directed therapeutic effects. The maturity of evidence differs substantially among immune-cell populations. Macrophages and T cells are the most extensively studied. For these cells, some studies have progressed from pathway-level validation to functional improvement in animal models, and limited clinical evidence is also available. In contrast, neutrophils and mast cells are supported mainly by preliminary intervention data, with most findings derived from *in vitro* experiments or single-animal-model studies. Direct validation using synovial tissue or synovial fluid samples remains insufficient. Thus, evidence related to these cells remains exploratory or auxiliary. Evidence for B cells is mainly limited to scattered findings based on peripheral blood immune markers, whereas evidence for DCs and NK cells is even more limited. At present, these three cell types are not sufficiently supported as independent therapeutic targets in natural-product-based OA interventions. Another important limitation is that most studies focus on a single immune-cell type. Few studies have examined how interactions among different immune-cell populations contribute to cartilage degradation, synovial inflammation, subchondral bone remodeling, and pain sensitization. This gap is important because immune dysregulation in OA is likely driven by networks of innate and adaptive immune responses rather than by a single isolated cell population.

Taken together, natural products should currently be viewed as promising candidates for OA immunomodulation, but not yet as proven disease-modifying strategies. The main contribution of this review is to evaluate differences in the strength and maturity of evidence across immune-cell populations using a cell-type-centered evidence maturity framework. Future studies should move beyond descriptive immune-marker changes and prioritize local joint validation, functional testing, intervention standardization, and phenotype-based patient stratification. Moreover, studies assessing multiple immune-cell populations within the same model may help clarify how natural products regulate immune-cell networks in OA joints. Such evidence will be essential for defining the clinical value of natural products and identifying the patient subgroups most likely to benefit from immune-modulating natural-product interventions.

## References

[1] Hunter DJ Bierma-Zeinstra S . Osteoarthritis. Lancet. (2019) 393:1745–59. doi: 10.1016/S0140-6736(19)30417-9 31034380

[2] Zhang Y Han Y Sun Y Hao L Gao Y Ye J . Osteoarthritis: molecular pathogenesis and potential therapeutic options. Signal Transduct Target Ther. (2026) 11:81. doi: 10.1038/s41392-025-02556-6 41781374 PMC12960730

[3] Zhu R Fang H Wang J Ge L Zhang X Aitken D . Inflammation as a therapeutic target for osteoarthritis: a literature review of clinical trials. Clin Rheumatol. (2024) 43:2417–33. doi: 10.1007/s10067-024-07042-y 38961031 PMC11269414

[4] Wen P Liu L . Functions of macrophages, T cells, and neutrophils in the synovial microenvironment of osteoarthritis. J Inflammation Res. (2025) 18:15671–86. doi: 10.2147/JIR.S563253 41243987 PMC12619582

[5] Lambert C Zappia J Sanchez C Florin A Dubuc JE Henrotin Y . The damage-associated molecular patterns (DAMPs) as potential targets to treat osteoarthritis: perspectives from a review of the literature. Front Med (Lausanne). (2021) 7:607186. doi: 10.3389/fmed.2020.607186 33537330 PMC7847938

[6] Millerand M Sudre L Nefla M Pène F Rousseau C Pons A . Activation of innate immunity by 14-3-3ϵ, a new potential alarmin in osteoarthritis. Osteoarthritis Cartilage. (2020) 28:646–57. doi: 10.1016/j.joca.2020.03.002 32173627

[7] Zhao K Ruan J Nie L Ye X Li J . Effects of synovial macrophages in osteoarthritis. Front Immunol. (2023) 14:1164137. doi: 10.3389/fimmu.2023.1164137 37492583 PMC10364050

[8] Zheng CQ Zeng LJ Liu ZH Miao CF Yao LY Song HT . Insights into the roles of natural killer cells in osteoarthritis. Immunol Invest. (2024) 53:766–87. doi: 10.1080/08820139.2024.2337025 38622991

[9] Hao G Han S Xiao Z Shen J Zhao Y Hao Q . Synovial mast cells and osteoarthritis: current understandings and future perspectives. Heliyon. (2024) 10:e41003. doi: 10.1016/j.heliyon.2024.e41003 39720069 PMC11665477

[10] Liu M Wu C Wu C Zhou Z Fang R Liu C . Immune cells differentiation in osteoarthritic cartilage damage: friends or foes? Front Immunol. (2025) 16:1545284. doi: 10.3389/fimmu.2025.1545284 40201177 PMC11975574

[11] Kaneva MK . Neutrophil elastase and its inhibitors-overlooked players in osteoarthritis. FEBS J. (2022) 289:113–6. doi: 10.1111/febs.16194 34580987

[12] Wen Z Qiu L Ye Z Tan X Xu X Lu M . The role of Th/Treg immune cells in osteoarthritis. Front Immunol. (2024) 15:1393418. doi: 10.3389/fimmu.2024.1393418 39364408 PMC11446774

[13] Burt KG Scanzello CR . B cells in osteoarthritis: simply a sign or a target for therapy? Osteoarthritis Cartilage. (2023) 31:1148–51. doi: 10.1016/j.joca.2023.06.002 37328048 PMC10680778

[14] Moulin D Sellam J Berenbaum F Guicheux J Boutet MA . The role of the immune system in osteoarthritis: mechanisms, challenges and future directions. Nat Rev Rheumatol. (2025) 21:221–36. doi: 10.1038/s41584-025-01223-y 40082724

[15] Sebastian A Hum NR McCool JL Wilson SP Murugesh DK Martin KA . Single-cell RNA-Seq reveals changes in immune landscape in post-traumatic osteoarthritis. Front Immunol. (2022) 13:938075. doi: 10.3389/fimmu.2022.938075 35967299 PMC9373730

[16] Fang S Zhang B Xiang W Zheng L Wang X Li S . Natural products in osteoarthritis treatment: bridging basic research to clinical applications. Chin Med. (2024) 19:25. doi: 10.1186/s13020-024-00899-w 38360724 PMC10870578

[17] Xu C Cui X Shi Y Zhang T Ni Z Li K . Natural products in the treatment of osteoarthritis: current status and prospects. J Orthop Translat. (2025) 55:94–120. doi: 10.1016/j.jot.2025.07.007 40995602 PMC12454292

[18] Lee YT Yunus MHM Ugusman A Yazid MD . Natural compounds affecting inflammatory pathways of osteoarthritis. Antioxidants (Basel). (2022) 11:1722. doi: 10.3390/antiox11091722 36139796 PMC9495743

[19] Maouche A Boumediene K Baugé C . Bioactive compounds in osteoarthritis: molecular mechanisms and therapeutic roles. Int J Mol Sci. (2024) 25:11656. doi: 10.3390/ijms252111656 39519204 PMC11546619

[20] Hu K Song M Song T Jia X Song Y . Osteoimmunology in osteoarthritis: unraveling the interplay of immunity, inflammation, and joint degeneration. J Inflammation Res. (2025) 18:4121–42. doi: 10.2147/JIR.S514002 40125089 PMC11930281

[21] Panichi V Costantini S Grasso M Arciola CR Dolzani P . Innate immunity and synovitis: key players in osteoarthritis progression. Int J Mol Sci. (2024) 25:12082. doi: 10.3390/ijms252212082 39596150 PMC11594236

[22] Kuang G Tan X Liu X Li N Yi N Mi Y . The role of innate immunity in osteoarthritis and the connotation of 'immune-joint' axis: a narrative review. Comb Chem High Throughput Screen. (2024) 27:2170–9. doi: 10.2174/0113862073264389231101190637 38243960

[23] Wang W Chu Y Zhang P Liang Z Fan Z Guo X . Targeting macrophage polarization as a promising therapeutic strategy for the treatment of osteoarthritis. Int Immunopharmacol. (2023) 116:109790. doi: 10.1016/j.intimp.2023.109790 36736223

[24] Zou X Xu H Qian W . Macrophage polarization in the osteoarthritis pathogenesis and treatment. Orthop Surg. (2025) 17:22–35. doi: 10.1111/os.14302 39638774 PMC11735378

[25] O'Brien K Tailor P Leonard C DiFrancesco LM Hart DA Matyas JR . Enumeration and localization of mesenchymal progenitor cells and macrophages in synovium from normal individuals and patients with pre-osteoarthritis or clinically diagnosed osteoarthritis. Int J Mol Sci. (2017) 18:774. doi: 10.3390/ijms18040774 28379175 PMC5412358

[26] Kraus VB McDaniel G Huebner JL Stabler TV Pieper CF Shipes SW . Direct *in vivo* evidence of activated macrophages in human osteoarthritis. Osteoarthritis Cartilage. (2016) 24:1613–21. doi: 10.1016/j.joca.2016.04.010 27084348 PMC4992586

[27] Utomo L Bastiaansen-Jenniskens YM Verhaar JA van Osch GJ . Cartilage inflammation and degeneration is enhanced by pro-inflammatory (M1) macrophages *in vitro*, but not inhibited directly by anti-inflammatory (M2) macrophages. Osteoarthritis Cartilage. (2016) 24:2162–70. doi: 10.1016/j.joca.2016.07.018 27502245

[28] Zhang S Chuah SJ Lai RC Hui JHP Lim SK Toh WS . MSC exosomes mediate cartilage repair by enhancing proliferation, attenuating apoptosis and modulating immune reactivity. Biomaterials. (2018) 156:16–27. doi: 10.1016/j.biomaterials.2017.11.028 29182933

[29] Edwards JP Zhang X Frauwirth KA Mosser DM . Biochemical and functional characterization of three activated macrophage populations. J Leukoc Biol. (2006) 80:1298–307. doi: 10.1189/jlb.0406249 16905575 PMC2642590

[30] Chaney S Vergara R Qiryaqoz Z Suggs K Akkouch A . The involvement of neutrophils in the pathophysiology and treatment of osteoarthritis. Biomedicines. (2022) 10:1604. doi: 10.3390/biomedicines10071604 35884909 PMC9313259

[31] Wang G Jing W Bi Y Li Y Ma L Yang H . Neutrophil elastase induces chondrocyte apoptosis and facilitates the occurrence of osteoarthritis via caspase signaling pathway. Front Pharmacol. (2021) 12:666162. doi: 10.3389/fphar.2021.666162 33935789 PMC8080035

[32] Sun GJ Xu F Jiao XY Yin Y . Advances in research of neutrophil extracellular trap formation in osteoarticular diseases. World J Orthop. (2025) 16:106377. doi: 10.5312/wjo.v16.i5.106377 40496259 PMC12146969

[33] Farinelli L Aquili A Mattioli-Belmonte M Manzotti S D'Angelo F Ciccullo C . Synovial mast cells from knee and hip osteoarthritis: histological study and clinical correlations. J Exp Orthop. (2022) 9:13. doi: 10.1186/s40634-022-00446-2 35079910 PMC8789998

[34] Kulkarni P Harsulkar A Märtson AG Suutre S Märtson A Koks S . Mast cells differentiated in synovial fluid and resident in osteophytes exalt the inflammatory pathology of osteoarthritis. Int J Mol Sci. (2022) 23:541. doi: 10.3390/ijms23010541 35008966 PMC8745477

[35] Wang Q Lepus CM Raghu H Reber LL Tsai MM Wong HH . IgE-mediated mast cell activation promotes inflammation and cartilage destruction in osteoarthritis. Elife. (2019) 8:e39905. doi: 10.7554/eLife.39905 31084709 PMC6516833

[36] Loucks A Maerz T Hankenson K Moeser A Colbath A . The multifaceted role of mast cells in joint inflammation and arthritis. Osteoarthritis Cartilage. (2023) 31:567–75. doi: 10.1016/j.joca.2023.01.005 36682447

[37] Ohashi Y Uchida K Fukushima K Satoh M Koyama T Tsuchiya M . NGF expression and elevation in hip osteoarthritis patients with pain and central sensitization. BioMed Res Int. (2021) 2021:9212585. doi: 10.1155/2021/9212585 34589551 PMC8476257

[38] Zhao X Younis S Shi H Hu S Zia A Wong HH . RNA-seq characterization of histamine-releasing mast cells as potential therapeutic target of osteoarthritis. Clin Immunol. (2022) 244:109117. doi: 10.1016/j.clim.2022.109117 36109004 PMC10752578

[39] Eitner A Rutte V Marintschev I Hofmann GO Schaible HG . Enhanced joint pain in diabetic patients with knee osteoarthritis is associated with increased synovitis, synovial immune cell infiltration, and erythrocyte extravasation. Front Endocrinol. (2024) 15:1477384. doi: 10.3389/fendo.2024.1477384 39469580 PMC11513275

[40] Jaime P García-Guerrero N Estella R Pardo J García-Álvarez F Martinez-Lostao L . CD56+/CD16- natural killer cells expressing the inflammatory protease granzyme A are enriched in synovial fluid from patients with osteoarthritis. Osteoarthritis Cartilage. (2017) 25:1708–18. doi: 10.1016/j.joca.2017.06.007 28668542

[41] Nie F Ding F Chen B Huang S Liu Q Xu C . Dendritic cells aggregate inflammation in experimental osteoarthritis through a toll-like receptor (TLR)-dependent machinery response to challenges. Life Sci. (2019) 238:116920. doi: 10.1016/j.lfs.2019.116920 31610189

[42] Rosshirt N Trauth R Platzer H Tripel E Nees TA Lorenz HM . Proinflammatory T cell polarization is already present in patients with early knee osteoarthritis. Arthritis Res Ther. (2021) 23:37. doi: 10.1186/s13075-020-02410-w 33482899 PMC7821658

[43] Sakkas LI Scanzello C Johanson N Burkholder J Mitra A Salgame P . T cells and T-cell cytokine transcripts in the synovial membrane in patients with osteoarthritis. Clin Diagn Lab Immunol. (1998) 5:430–7. doi: 10.1128/CDLI.5.4.430-437.1998 9665944 PMC95595

[44] Sohn HS Choi JW Jhun J Kwon SP Jung M Yong S . Tolerogenic nanoparticles induce type II collagen-specific regulatory T cells and ameliorate osteoarthritis. Sci Adv. (2022) 8:eabo5284. doi: 10.1126/sciadv.abo5284 36427299 PMC9699678

[45] Xiao J Zhang P Cai FL Luo CG Pu T Pan XL . IL-17 in osteoarthritis: a narrative review. Open Life Sci. (2023) 18:20220747. doi: 10.1515/biol-2022-0747 37854319 PMC10579884

[46] Shiokawa S Matsumoto N Nishimura J . Clonal analysis of B cells in the osteoarthritis synovium. Ann Rheum Dis. (2001) 60:802–5. doi: 10.1136/ard.60.8.802 11454647 PMC1753802

[47] Lu P Li Y Yang S Yao H Tu B Ning R . B cell activation, differentiation, and their potential molecular mechanisms in osteoarthritic synovial tissue. J Inflammation Res. (2025) 18:2137–51. doi: 10.2147/JIR.S503597 39959649 PMC11829641

[48] Zhu W Zhang X Jiang Y Liu X Huang L Wei Q . Alterations in peripheral T cell and B cell subsets in patients with osteoarthritis. Clin Rheumatol. (2020) 39:523–32. doi: 10.1007/s10067-019-04768-y 31624962

[49] Sahu N Bedi YS Grandi F Maloney WJ Chu CR Bhutani N . Multiparametric profiling of circulating immune cells identifies an expansion of CD25high switched memory B cells in osteoarthritis. Arthritis Rheumatol. (2025) 77:1228–41. doi: 10.1002/art.43186 40229860 PMC12353273

[50] Deng Z Zhang Q Zhao Z Li Y Chen X Lin Z . Crosstalk between immune cells and bone cells or chondrocytes. Int Immunopharmacol. (2021) 101:108179. doi: 10.1016/j.intimp.2021.108179 34601329

[51] Wang M Tan G Jiang H Liu A Wu R Li J . Molecular crosstalk between articular cartilage, meniscus, synovium, and subchondral bone in osteoarthritis. Bone Joint Res. (2022) 11:862–72. doi: 10.1302/2046-3758.1112.BJR-2022-0215.R1 36464496 PMC9792876

[52] Zhang K Wang Z He J Lu L Wang W Yang A . Mechanisms of synovial macrophage polarization in osteoarthritis pathogenesis and their therapeutic implications. Front Immunol. (2025) 16:1637731. doi: 10.3389/fimmu.2025.1637731 41376619 PMC12685870

[53] Fu HY Jie LS Gong ZJ Huang ZL Zhu ZS Liu JY . Mechanism study on liquiritin alleviating nociceptive sensitization in knee osteoarthritis via promoting M2 macrophage polarization through regulation of the Rap1/PI3K/Akt signaling pathway. Phytother Res. (2026) 40:701–20. doi: 10.1002/ptr.70174 41494619

[54] Sun Q Nan XY Wang H Pan S Ji G Guo YF . Polydatin retards the progression of osteoarthritis by maintaining bone metabolic balance and inhibiting macrophage polarization. Front Bioeng Biotechnol. (2025) 12:1514483. doi: 10.3389/fbioe.2024.1514483 39840130 PMC11747576

[55] Tsai PW Lee YH Chen LG Lee CJ Wang CC . *In vitro* and *in vivo* anti-osteoarthritis effects of 2,3,5,4'-tetrahydroxystilbene-2-O-β-d-glucoside from Polygonum multiflorum. Molecules. (2018) 23:571. doi: 10.3390/molecules23030571 29510478 PMC6017566

[56] Li W He H Du M Gao M Sun Q Wang Y . Quercetin as a promising intervention for rat osteoarthritis by decreasing M1-polarized macrophages via blocking the TRPV1-mediated P2X7/NLRP3 signaling pathway. Phytother Res. (2024) 38:1990–2006. doi: 10.1002/ptr.8158 38372204

[57] Ji X Du W Che W Wang L Zhao L . Apigenin inhibits the progression of osteoarthritis by mediating macrophage polarization. Molecules. (2023) 28:2915. doi: 10.3390/molecules28072915 37049677 PMC10095825

[58] Wang W Li T Zhang J Guan T Xu S Lai Y . Nystose mitigates mono sodium iodoacetate-induced knee osteoarthritis by inhibiting the NLRP3 inflammasome. Phytomedicine. (2026) 150:157598. doi: 10.1016/j.phymed.2025.157598 41338111

[59] Wu SQ Otero M Unger FM Goldring MB Phrutivorapongkul A Chiari C . Anti-inflammatory activity of an ethanolic Caesalpinia sappan extract in human chondrocytes and macrophages. J Ethnopharmacol. (2011) 138:364–72. doi: 10.1016/j.jep.2011.09.011 21963554 PMC3282169

[60] Peng L Xie Z Pei J Wang B Gao Y Qu Y . Puerarin alters the function of monocytes/macrophages and exhibits chondroprotection in mice. Mol Med Rep. (2019) 19:2876–82. doi: 10.3892/mmr.2019.9936 30720093

[61] Poursamimi J Shariati-Sarabi Z Tavakkol-Afshari J Mohajeri SA Ghoryani M Mohammadi M . Immunoregulatory effects of Krocina™, a herbal medicine made of crocin, on osteoarthritis patients: a successful clinical trial in Iran. Iran J Allergy Asthma Immunol. (2020) 19:253–63. doi: 10.18502/ijaai.v19i3.3453 32615659

[62] Atabaki M Shariati-Sarabi Z Tavakkol-Afshari J Mohammadi M . Significant immunomodulatory properties of curcumin in patients with osteoarthritis; a successful clinical trial in Iran. Int Immunopharmacol. (2020) 85:106607. doi: 10.1016/j.intimp.2020.106607 32540725

[63] Sun MD Shang XL Wang XJ . Tanshinone IIA regulates Th17/Treg immune balance and affects TLR4-related pathway expression in rats with osteoarthritis cartilage degeneration. J Med Mol Biol. (2024) 21:521–9. doi: 10.3870/j.issn.1672-8009.2024.06.004

[64] Mahmoud FF Al-Awadhi AM Haines DD . Amelioration of human osteoarthritis symptoms with topical 'biotherapeutics': a phase I human trial. Cell Stress Chaperones. (2015) 20:267–76. doi: 10.1007/s12192-014-0553-0 25427747 PMC4326390

[65] Jhun J Min HK Na HS Kwon JY Ryu J Cho KH . Combinatorial treatment with Lactobacillus acidophilus LA-1, vitamin B, and curcumin ameliorates the progression of osteoarthritis by inhibiting the pro-inflammatory mediators. Immunol Lett. (2020) 228:112–21. doi: 10.1016/j.imlet.2020.10.008 33137380

[66] Amirova KM Dimitrova PA Leseva MN Koycheva IK Dinkova-Kostova AT Georgiev MI . The triterpenoid Nrf2 activator, CDDO-Me, decreases neutrophil senescence in a murine model of joint damage. Int J Mol Sci. (2023) 24:8775. doi: 10.3390/ijms24108775 37240121 PMC10217923

[67] Cross AL Hawkes J Wright HL Moots RJ Edwards SW . APPA (apocynin and paeonol) modulates pathological aspects of human neutrophil function, without supressing antimicrobial ability, and inhibits TNFα expression and signalling. Inflammopharmacology. (2020) 28:1223–35. doi: 10.1007/s10787-020-00715-5 32383062 PMC7525285

[68] Ortega-Collazos E Otero E López-Jurado C Navarro C Martín-Cordero L Gálvez I . Neuroimmunomodulation induced by mud-bath therapy: clinical benefits and bioregulation of the innate/inflammatory responses induced by a peloid enriched with rosmarinic acid in elderly patients with osteoarthritis. Int J Biometeorol. (2025) 69:2115–24. doi: 10.1007/s00484-025-02984-7 40668398 PMC12479573

[69] Li J Song D Li B Wang Y Sun H Li Q . Exploring the mechanism of tetramethylpyrazine in the treatment of osteoarthritis based on network pharmacology. Front Chem. (2024) 12:1415390. doi: 10.3389/fchem.2024.1415390 39539395 PMC11557414

[70] Yoo JM Yang JH Kim YS Yang HJ Cho WK Ma JY . Inhibitory effects of Viscum coloratum extract on IgE/antigen-activated mast cells and mast cell-derived inflammatory mediator-activated chondrocytes. Molecules. (2016) 22:37. doi: 10.3390/molecules22010037 28036032 PMC6155826

[71] Cordaro M Siracusa R Impellizzeri D D'Amico R Peritore AF Crupi R . Safety and efficacy of a new micronized formulation of the ALIAmide palmitoylglucosamine in preclinical models of inflammation and osteoarthritis pain. Arthritis Res Ther. (2019) 21:254. doi: 10.1186/s13075-019-2048-y 31779692 PMC6883534

[72] Penatti A Facciotti F De Matteis R Larghi P Paroni M Murgo A . Differences in serum and synovial CD4+ T cells and cytokine profiles to stratify patients with inflammatory osteoarthritis and rheumatoid arthritis. Arthritis Res Ther. (2017) 19:103. doi: 10.1186/s13075-017-1305-1 28526072 PMC5437517

[73] Rosshirt N Hagmann S Tripel E Gotterbarm T Kirsch J Zeifang F . A predominant Th1 polarization is present in synovial fluid of end-stage osteoarthritic knee joints: analysis of peripheral blood, synovial fluid and synovial membrane. Clin Exp Immunol. (2019) 195:395–406. doi: 10.1111/cei.13230 30368774 PMC6378378

[74] Michoel T Zhang JD . Causal inference in drug discovery and development. Drug Discov Today. (2023) 28:103737. doi: 10.1016/j.drudis.2023.103737 37591410

[75] Danzeng L Sun Y He Z Hou X Li L . Single-cell sequencing reveals the immune microenvironment in osteoarthritis: from heterogeneity to therapeutic targets. Int Immunopharmacol. (2025) 165:115521. doi: 10.1016/j.intimp.2025.115521 40934543

[76] Li L Fan X Crawford R Mao X Ong LJY Gao F . Understanding pain heterogeneity in osteoarthritis patients: a narrative review. Front Med. (2025) 19:769–88. doi: 10.1007/s11684-025-1143-5 40715664

[77] He Z Hao H Chen B Li X Mao D Jin Y . Pathogenesis and therapeutic strategies of osteoarthritis: roles of immune cells, inflammatory mediators, and pathogenic signaling pathways. Immun Inflammation. (2026) 2:3. doi: 10.1007/S44466-025-00022-0 30311153

[78] Wang C Zeng Z Wang T Xie Z Zhang J Dong W . Unraveling the spatial and signaling dynamics and splicing kinetics of immune infiltration in osteoarthritis synovium. Front Immunol. (2025) 16:1521038. doi: 10.3389/fimmu.2025.1521038 40181977 PMC11966058

[79] Heinrich M Jalil B Abdel-Tawab M Echeverria J Kulić Ž McGaw LJ . Best Practice in the chemical characterisation of extracts used in pharmacological and toxicological research-The ConPhyMP-Guidelines. Front Pharmacol. (2022) 13:953205. doi: 10.3389/fphar.2022.953205 36176427 PMC9514875

[80] Mobasheri A Loeser R . Clinical phenotypes, molecular endotypes and theratypes in OA therapeutic development. Nat Rev Rheumatol. (2024) 20:525–6. doi: 10.1038/s41584-024-01126-4 38760581

[81] Roman-Blas JA Mendoza-Torres LA Largo R Herrero-Beaumont G . Setting up distinctive outcome measures for each osteoarthritis phenotype. Ther Adv Musculoskelet Dis. (2020) 12:1759720X20937966. doi: 10.1177/1759720X20937966 32973934 PMC7491224

[82] Rahmani J Manzari N Thompson J Clark CCT Villanueva G Varkaneh HK . The effect of saffron on weight and lipid profile: A systematic review, meta-analysis, and dose-response of randomized clinical trials. Phytother Res. (2019) 33:2244–55. doi: 10.1002/ptr.6420 31264281

[83] Ye Q Zhou K Gao Q Li C Sheng Y Zou L . Prevention and treatment of knee osteoarthritis by natural products: potential mechanisms based on articular cartilage targets. Chin J Nat Med. (2026) 24:129–44. doi: 10.1016/S1875-5364(26)61097-7 41708246

[84] Peng Z Li Y Sun N Jia Y Shi M Xi K . Advancements in osteoarthritis treatment: The potential of natural herbs in reducing inflammation and cartilage degeneration. Phytother Res. (2026) 40:1603–25. doi: 10.1002/ptr.70222 41587818

[85] Lu Y Zhou H Luo H Hu W Hu L Xie J . Molecular mechanisms of traditional Chinese medicine in treating osteoarthritis. Am J Chin Med. (2026) 54:763–93. doi: 10.1142/S0192415X2650028X 42010988

